# Strong and weak polarization-dependent interactions in connected and disconnected plasmonic nanostructures†

**DOI:** 10.1039/d1na00620g

**Published:** 2022-01-10

**Authors:** Damien Eschimèse, François Vaurette, Céline Ha, Steve Arscott, Thierry Mélin, Gaëtan Lévêque

**Affiliations:** Univ. Lille, CNRS, Centrale Lille, Junia, Univ. Polytechnique Hauts-de-France, UMR 8520 – IEMN – Institut d’Electronique de Microélectronique et de Nanotechnologie F-59000 Lille France gaetan.leveque@univ-lille.fr

## Abstract

We explore numerically and experimentally the formation of hybridized modes between a bright mode displayed by a gold nanodisc and either dark or bright modes of a nanorod – both elements being either separated by a nanometer-size gap (disconnected system) or relied on a metal junction (connected system). In terms of modeling, we compare the scattering or absorption spectra and field distributions obtained under oblique-incidence plane wave illumination with quasi-normal mode computation and an analytical model based on a coupled oscillator model. Both connected and disconnected systems have very different plasmon properties in longitudinal polarization. The disconnected system can be consistently understood in terms of the nature of hybridized modes and coupling strength using either QNMs or coupled oscillator model; however the connected configuration presents intriguing peculiarities based on the strong redistribution of charges implied by the presence of the metal connection. In practice, the fabrication of disconnected or connected configurations depends on the mitigation of lithographic proximity effects inherent to top-down lithography methods, which can lead to the formation of small metal junctions, while careful lithographic dosing allows one to fabricate disconnected systems with a gap as low as 20 nm. We obtained a very good agreement between experimentally measured scattering spectra and numerical predictions. The methods and analyses presented in this work can be applied to a wide range of systems, for potential applications in light–matter interactions, biosensing or strain monitoring.

## Abstract 2

Left: electric field amplitude (color) and surface charge amplitude (grayscale) of the low-energy quasi-normal modes of the disconnected (top) and connected (bottom) nanostructures. Right: SEM images of the experimental nanostructures.

## Graphical abstract

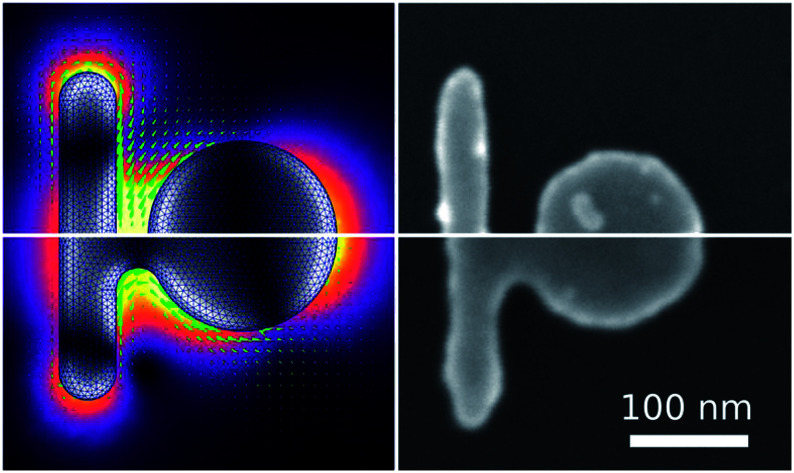

## Introduction

Fano profiles in coupled metal nanoparticles displaying localized surface plasmon (LSP) modes have been investigated in recent years in view of potential applications, such as biosensing.^1,2^ Following theoretical predictions,^3^ Fano resonance has been observed in various types of structures such as *inter alia*: nanoclusters,^4^ plasmonic particles involving nanorods, metal–insulator–metal systems,^5,6^ and between interconnected metal structures.^7^ A fundamental condition for the observation of a Fano profile is the interference between spectrally-overlapping broad and discrete modes.^8^ As so-called dark-modes display weak scattering losses, they generally play the role of narrow resonance, while the broad one results from the excitation of a bright mode with a strong dipolar component. However, Fano profiles can also be obtained between bright modes, as has been investigated theoretically by Forestiere *et al.* using a quasi-electrostatic approach,^9^ or experimentally and numerically by Lovera *et al.*^10^

Changing the gap which separates the nanoparticles allows tuning the system between weak and strong coupling. For large gaps, each nanoparticle maintains its own modal properties, but below a critical value,^11^ individual modes hybridize and Fano resonances result from interference between sub- and super-radiant collective modes. Strong coupling in plasmonics has been investigated between metal particles and Fabry–Pérot resonators,^12^ propagating surface plasmons,^13,14^ localized waveguide resonances,^15^ and quantum emitters.^11,14,16^ Besides, as metal nanorods display an arbitrarily large number of alternating bright and dark modes depending on their length, they have frequently been used to investigate Fano resonances and strong coupling, combined in linear arrangements,^17,18^ “dolmen-type” assemblies,^19,20^ T-shaped dimers,^21^ H-structures,^22^ χ-shaped structures,^23^ or coupled to nanospheres.^24^

In both strong and weak coupling, information about interfering modes can be obtained by comparing extinction spectra with *ad hoc* fitting equations,^1,21^ coupled-oscillator models,^10^ and analytical expressions based on electrostatic approximation.^23^ However, direct numerical access to the so-called quasi-normal modes (QNMs) displayed by either individual or coupled systems has been made possible recently, allowing one to obtain essential information on pure eigenmodes—such as resonance wavelengths or widths—without having to deal with the problem of mode overlapping and complex illumination conditions necessary to break unwanted symmetry properties.^25–27^ Strong coupling has been investigated using QNM simulations in a variety of systems with interacting metal nanoparticles.^28–31^

In this article, we investigate the transition between weak and strong coupling regimes in the electromagnetic interaction between metallic nanorods (NRs) and metallic nanodisks (NDs) in physical proximity or conductive contact. We combine standard scattering and absorption spectra computation under plane wave illumination and QNM simulations based on a home-made iterative procedure allowing one to compute complex wavenumbers. The modeling allows deep physical insight and several predictions to be made concerning connected and disconnected NR–ND nanostructures. Practical gold NR–ND nanostructures are fabricated on silicon wafers using electron beam lithography for lift-off patterning of metallization, and are subsequently characterized optically using an in-house characterization bench.^32^ The measurements are then compared with the predictions of the modeling.

## Numerical investigation

**Fig. 1:**
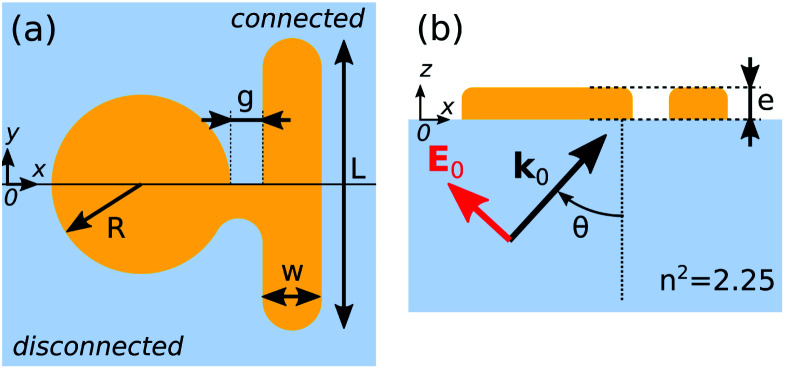
Schematic of the investigated nanostructure: (a) top-view of the disconnected (top) and connected (bottom) configurations; (b) side-view in the incidence plane for p polarization.

The NR is parallel to the *Oy* axis and has a width *w* = 40 nm and a varying length *L*, while the ND has a radius *R* = 66 nm, both having a thickness *e* = 30 nm and lying on a silica substrate with a refractive index *n* = 1.5. Two configurations are considered. In the hereafter called “disconnected” case, (Fig. 1a, top), both components are separated by a gap *g*. Its value will be either varied or fixed to a reference value of *g* = 22 nm. In the “connected” system, (Fig. 1a, bottom), the gap is filled with a small bridge of gold with length 22 nm. In both situations, the structure is illuminated from the glass substrate by a plane wave with wavenumber **k**_0_ parallel to the (*xz*) planes, the polarization being either p (**H**_0_∥*y*) or s (**E**_0_∥*y*), Fig. 1(b). The incidence angle *θ* is set to a reference value of 45° in the following, unless otherwise specified.

Numerical simulations were done with open-source software: both plane wave illumination and quasi-normal-mode finite element simulations have been performed using FreeFem++,^33^ after elaboration of the geometry and the generation of a mesh using Gmsh.^34^ Details on the numerical procedure are supplied in ESI, Section 1.† Scattering and absorption spectra of the isolated nanodisk and two nanorods with different lengths are plotted in Fig. S3.† QNM simulations indicate that the ND alone has a 114 nm-wide dipolar resonance centered at 682 nm. The 84 nm-long NR shows one resonance close to 645 nm with width 48 nm, corresponding to the lowest order bright mode, while the 244 nm-long NR shows a maximum close to 715 nm with width 37 nm corresponding to the lowest order dark mode. In the following, the order of NR modes will indicate the number of surface charges nodes dsitributed along its axis: odd modes are bright, while even modes are dark.

### Disconnected nanorod–nanodisk

**Fig. 2:**
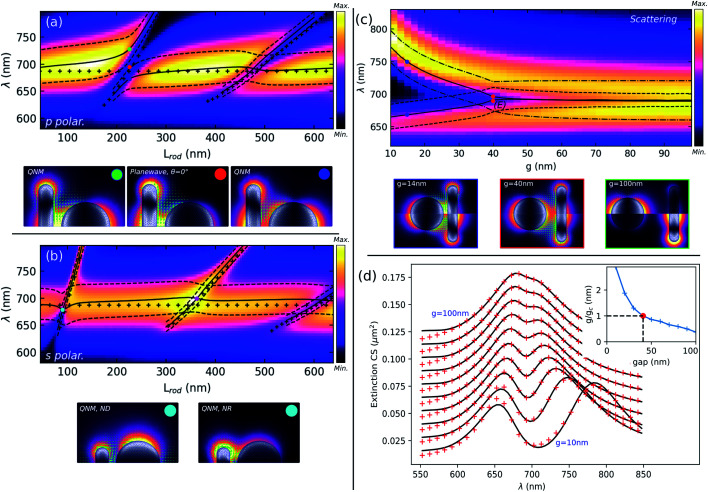
Numerical modeling of the disconnected system. Left panel shows the effect of the nanorod length *L*: (a) scattering spectrum for p polarization and (b) for s polarization, compared with QNM wavelengths (solid lines) and widths (dashed lines, separated by half the full width at half maxium for readability). For each polarization, QNMs are plotted for selected parameters indicated by colored dots. Colormaps show the intensity of the field 10 nm above the interface, whereas the amplitude of the surface charges is plotted in grayscale on the particle surface. The right panel shows the effect of the gap variation. (c) Scattering spectrum as a function of the gap for p polarization, and QNMs for wavelengths and gaps indicated by the colored dots (bottom). (d) Fit of the extinction spectra with a coupled oscillator model for a gap varying from 10 nm (bottom) to 100 nm (top) with a 10 nm step. Inset: evolution of the normalized coupling coefficient with the gap.

Bright and dark modes of the NR are susceptible to interact with the ND dipolar mode when the two components are placed close to each other. In this configuration, computed scattering spectra presented in Fig. 2 differ a lot with polarization. Indeed, as the incidence plane (*Oxz*) is perpendicular to the NR axis, see Fig. 1, the incident field imposes a symmetry selection rule on modes excited inside each nanoparticle: for p (resp. s) incident polarization, only NR dark (resp. bright) modes can be excited, together with the *x*-oriented (resp. *y*) dipolar mode of the ND. For p polarization, the excitation of dark modes is mediated by the dipolar mode of the nanodisk, which acts as a secondary localized source facing the center of the nanorod.

Fig. 2(a) and (b) show the effect of varying the nanorod length *L*. Fig. 2(a) and (b) show scattering spectra in color, respectively, for p and s polarizations, together with the wavelengths of the QNMs in black solid lines, and the dashed lines indicate the resonance width of each mode extracted from the imaginary part of the complex eigenfrequencies. The wavelengths of the uncoupled ND and NR are indicated by black crosses. Below each scattering spectrum are plotted field distributions under plane wave illumination or associated with QNMs, for parameters indicated by colored dots in the spectra.

In p polarization, scattering spectra in Fig. 2(a) show clear minima for specific ranges of NR lengths where its second (*L* ≈ 220 nm) and fourth order (*L* ≈ 480 nm) dark modes are close to the ND resonance. If this behaviour suggests a Fano profile resulting from interferences between the NR dark modes and the ND bright mode, QNM simulations clearly indicate a different type of interaction between the shorter and longer NR: a mode anti-crossing is obtained, as shown in Fig. 2(a), for the second order mode, while a mere overlapping occurs for the fourth order mode. This is in agreement with the scattering spectra presented in Fig. S4(a),† where a minimum absorption is obtained close to the middle of the anti-crossing at *L* = 220 nm, while a maximum occurs close to *L* = 480 nm. Hence, the ND dipolar mode and the NR second order dark mode splits into two branches whose widths rapidly change close to the anti-crossing, demonstrating a plasmonic strong coupling. Field distributions for *L* = 228 nm show light enhancement within the gap only for the lower branch, which then corresponds to the bonding combination of the NR second order dark mode and the ND dipole mode, while the upper branch is associated with the anti-bonding mode. Note that the term “bonding” refers to the fact that the surface charges are of opposite sign on both sides of the gap, which implies an attractive and then a negative electrostatic interaction term which lowers the energy of that mode. The anti-bonding mode has charges of the same sign inside the gap, implying a repulsive positive term which increases its energy. The red dot marks the wavelength where minimum scattering is reached for this NR length: the corresponding field distribution computed for plane wave illumination shows a low field inside the ND compared to the NR, as the field scattered by the nanodisk is mostly absorbed by the NR. Finally, when the fourth-order dark mode interacts with the ND dipole (close to *L* = 480 nm), both modes are slightly shifted but no hybridization occurs: the Fano-type asymmetric profiles observed in the scattering spectra are a sign of interferences between unhybridized NR dark and ND bright plasmon modes.

For s polarization, the incident field can only excite antisymmetrical modes with respect to (*Oxz*), which are odd-parity bright modes of the NR, and the *y*-oriented dipolar mode of the ND. In Fig. 2(b), an obvious difference with the p polarization is that no anti-crossing can be observed. QNM calculations show that a simple mode crossing is obtained when the NR length is close to 92 nm. For this parameter, the wide dipolar mode associated with the ND weakly interacts with the first NR bright mode, which mostly results in a small blue shift of the ND mode, without a noticeable change of the width. Field distributions of both QNMs at the crossing point, indicated by a cyan dot, show that the light remains mostly confined close to the NR for the narrow mode, while the wide mode, despite being localized mostly around the ND, still has a significant contribution close to the NR. However, interference occurs between the field scattered by the nanorod and the disk, as can be seen in Fig. 2(b), resulting in either reduced (*L* = 92 nm) or enhanced (*L* = 360 nm) scattering, whereas the opposite is obtained for absorption, Fig. S4(b).†

We finally present the influence of the gap on the coupling between the ND dipolar mode and the NR second order (dark) mode, in the case of p polarization. Fig. 2(c) shows the effect of the gap *g* on the scattering spectra, together with the position and width of the QNMs, when the ND interacts with a 228 nm-long NR. For this particular value of *L*, the resonance wavelengths of the isolated NR and ND are almost identical as can be verified for *g* = 100 nm. This simulation clearly evidences a bifurcation point (*E*) close to *g* = 40 nm, below which the NR dark mode and the ND dipolar mode start to hybridize into bonding and antibonding modes with comparable widths, while the system enters into a strong coupling regime. Movie S1 in the ESI† clearly shows the abrupt change in the field distribution on both sides of the bifurcation point. This is illustrated by field maps in Fig. 2(c), where the top half of each plot corresponds to the upper branch (bonding mode and ND dipolar mode) and the bottom half shows the lower branch (anti-bonding mode and NR dark mode). The blue dot indicates a gap of *g* = 14 nm, where a clear distinction between field distributions of the bonding and anti-bonding modes is obtained, similar to the above discussion. At the bifurcation point (red dot), both field maps are quasi indistinguishable, as are the wavelength and width of both modes. For a large gap (green dot), field distributions and complex wavenumbers are consistent with the isolated particle properties. Note that a strong coupling is obtained (not shown) at the second crossing close to *L* = 480 nm, for a gap lower than 8 nm.

The transition from a weak to strong coupling regime generally occurs when the coupling strength between the two oscillators exceeds the losses.^10,11,35^ In order to assess the coupling between the two interacting modes, we have used a coupled oscillator model given by the following set of equations:^36^

1 *ẍ*_b_ + *γ*_b_*ẋ*_b_ + *ω*_b_^2^*x*_b_ + *gx*_d_ = *f* exp(−i*ωt*)*ẍ*_d_ + *γ*_d_*ẋ*_d_ + *ω*_d_^2^*x*_d_ + *gx*_b_ = 0

where *x*_b_ and *x*_d_ are the responses of the so-called diabatic (*i.e.* uncoupled) bright and dark modes, respectively, *ω*_d_ and *ω*_d_ are the associated resonance frequencies, *γ*_b_ and *γ*_d_ are the losses and *g* is the coupling parameter. The incident plane wave has an amplitude *f* and is only coupled to the bright mode. The analytical resolution of the system of eqn (1) allows expressing the amplitude of the bright mode dipole as *x*_b_(*ω*) = (*ω*_d_^2^ − *ω*^2^ − i*γ*_d_*ω*)/[(*ω*_d_^2^ − *ω*^2^ − i*γ*_d_*ω*)(*ω*_d_^2^ − *ω*^2^ − i*γ*_d_*ω*) − *g*^2^]. The imaginary part of *x*_b_(*ω*) has been used to fit the numerical extinction spectra. When *ω*_b_ = *ω*_d_ = *ω*_0_, the critical value of *g* between the weak and strong coupling is simply expressed as 
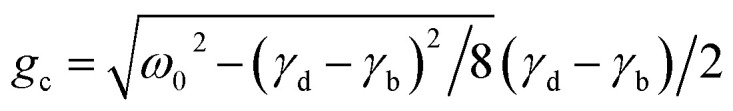
. Fig. 2(d) and S5† show the evolution of the fit with the gap variation. Diabatic modes have a weakly varying width with the gap, consistent with values found for isolated particles (see Section 2 in the ESI†). The red-shift of their resonance wavelengths for small gaps is attributed to the screening effect induced by the proximity of the other nanoparticles.^37^ The consistency of this approach is demonstrated by the fact that for a gap of 40 nm, which corresponds to the weak to strong coupling transition as found with QNM simulations, the normalized coupling coefficient *g*/*g*_c_ is very close to 1. Similar calculations performed as a function of the nanorod length for *g* = 22 nm show that the coupled oscillator model predicts as well a strong coupling close to *L* = 235 nm (*g*/*g*_c_ ≈ 1.6, Fig. S6†), while close to *L* = 480 nm the weak coupling is confirmed by the value *g*/*g*_c_ ≈ 0.75, lower than 1, Fig. S7.†

### Connected nanorod–nanodisk

We examine now the case where the nanocylinder and the nanorod are connected by a small conductive bridge, the previous 22 nm gap being filled with gold. This situation is different from the direct contact between the ND and the NR, *g* = 0 nm, which is anyway a mathematical limitation realized along a vertical line and which never occurs experimentally. Moreover, it is known that for gaps below a few nanometers, non-locality must be taken into account in the response of the metal, while quantum effects can occur for gaps below 1 nm, with important impacts on the resonance wavelengths and enhancement factors.^38,39^

**Fig. 3:**
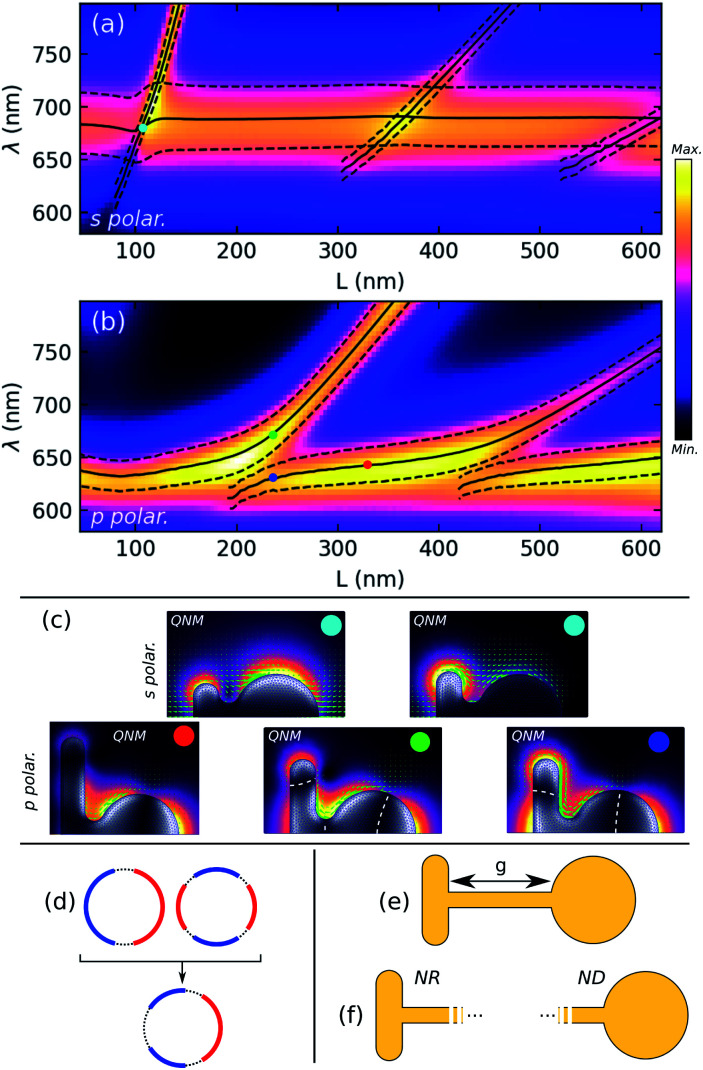
Numerical modeling of the connected system. (a) Scattering spectrum for p polarization and (b) for s polarization, compared with QNM wavelengths (solid lines) and widths (dashed lines). (c) QNMs for s and p polarizations (see Fig. 2). The white dashed lines indicate the areas where surface charges cancel. (d) Surface charge distributions for the ND mode (red dot on (b)) resulting from the hybridization between a dipolar and a quadripolar mode. (e) System connected by a wire of varying lengths and (f) NR or ND connected to an infinitely long wire.

Fig. 3 shows the results of the simulated scattering spectra for s, (a), and p polarization, (b), of the incident plane wave, together with the QNM wavelengths and widths, and selected field distributions, (c). Almost no differences with the disconnected nanostructures are observed in the scattering spectra for s polarization, where two surface plasmon modes with dipolar field distributions are identified: the wider mode, with a resonance wavelength of about 680 nm, is localized mostly on the disk, while the sharper mode with a *L*-dependent wavelength is localized on the rod, see Fig. 3(c), top. As for the disconnected system, there are particular values of *L* for which both modes are degenerate, close to *L* = 105 nm and *L* = 460 nm, with a slight blue-shift of the larger mode close to the first intersection. Overall, the similarity to the disconnected system for s polarization directly results from the fact that, because surface charges cancel in the incidence plane (which is an anti-symmetry plane for the system), the associated plasmon modes barely “see” the gap and the resulting spectra are mostly unchanged by the metal connection. This can be verified by the Movie S2 in the ESI† which compares the QNM field distributions for disconnected and connected nano-objects in s polarization. However, the resonances of the connected system are blue-shifted compared to the disconnected system, which can be explained by a reduction of the effective length of the rod due to the expulsion of the surface charges from the gap.

In p polarization, Fig. 3(b), the situation is very different as surface charges, which in the disconnected system are for example enhanced in the gap area for the bonding mode, are completely redistributed in the presence of the metal connection, with dramatic effects on the plasmonic properties. The scattering and absorption (see Fig. S8†) spectra show a succession of branches well separated by anti-crossings close to *L* = 240 nm and *L* = 460 nm. Again, QNM simulations help in understanding the shape of the associated surface plasmon modes. In the flattened portion of the branches, the field is mostly enhanced on the nanodisk (Fig. 3(c), red dot), and the width reaches 0.16 μm^−1^ for a wavelength of 644 nm, much lower than the resonance wavelength of the isolated ND. The field is localized on the NR part in the oblique branches, and the resonance width is about 0.12 μm^−1^ for *L* ∼ 350 nm, where the resonance wavelength is 790 nm. These values are to be compared with 0.24 μm^−1^ for the isolated nanodisk and 0.096 μm^−1^ for the isolated nanorod with the same resonance wavelength. Hence, far from anti-crossings, the ND (resp. NR) plasmon mode is narrower (resp. larger) in the connected system than the disconnected system. A simple qualitative interpretation can be given in both cases. For the ND plasmon mode, the surface charge distribution cancels in the metal connection, which is consistent with the superposition of a longitudinal dipole and a lower-wavelength, narrower (because dark), quadrupolar mode, as shown in Fig. 3(d): charges are then expelled from the gap region, the resonance wavelength is blue shifted and the reduced scattering losses make the mode narrower. In the oblique branches, the field is localized on the NR; however the mirror symmetry of the mode along the NR axis is broken in the presence of the metal connection, resulting in a non-zero net dipolar momentum along the (*Ox*) direction: scattering losses are then increased and the mode is wider. Similarly in s polarization, the resonance wavelength is blue-shifted due to reduced effective length of the NR related to the connection. Close to the first anti-crossing, *L* = 240 nm, the low-energy mode (green dot) is characterized by a field enhanced on the connection and shows three nodal lines in the surface charge distribution (white dashed lines) with one in the middle of the metal connection: like the disconnected system, the lowest frequency mode has charges of opposite sign on both edges of the metal bridge. The second mode (blue dot) has no charge cancellation in this area, consistently leading to a higher frequency due to the resulting repulsive interaction. Movie S3 included in the ESI† shows the QNM field and surface charge distribution as a function of the NR length in p polarization for both connected and disconnected configurations.

Similarly in the case of the disconnected system, we can wonder whether it is possible to use a coupled oscillator model to fit the extinction or scattering spectra obtained above in p polarization, in order to describe the plasmon modes of the connected system as the interaction between two unhybridized or diabatic modes and recover a coupling parameter. However, the fact that both modes are radiative, as discussed above, makes the model described using eqn (1) inapplicable as both diabatic modes are excited by the incident plane wave and interfere in the scattering spectra. As a consequence, a more general model like the one used by Lovera *et al.*^10^ could be used, but at the cost of three additional parameters: the polarisabilities of the diabatic modes and the distance between them. In practice, the large number of free parameters implies that a good enough fit of the numerical scattering spectra can be obtained for very different sets of diabatic modes (wavelength and width), and does not give further physical insight. Finally, when compared to the disconnected case, it is obvious that it is not possible anymore to easily describe the plasmon modes of the connected nanostructure as an interaction between the two modes of the uncoupled NR and ND. But in the light of the previous discussion, the effect of the metal junction can be seen as (i) expelling the surface charges from the contact area and (ii) inducing a coupling between the two halves of the structures. Diabatic modes would be the result of (i), and the exact numerical simulation of these uncoupled modes is not obvious. The nature of these modes can be however guessed by considering that the connected system investigated in this work is the short length limit of a NR and a ND connected by a metal wire of length *g*, see Fig. 3(e). In this model, the unhybridized modes would be associated with a NR or a ND connected to an infinitely long wire as shown in Fig. 3(f). The full investigation of this mechanism is however beyond the purpose of this paper.

## Experimental methods and results

### Top-down nanofabrication

Individual, stand-alone metallic nanostructures can be precisely patterned glass substrates using a combination of electron beam (eBeam) lithography, evaporation of thin film metals, and lift-off processes.^40,41^ Such structures have been optically-characterized.^32^ However, Fano-type structures require the precise tailoring of multiple nanometer-sized metallic mesa features. In addition to this, the small spacing between the structures must be accurately and precisely controlled as the gap governs the electromagnetic coupling and hence the overall behavior of the structure. These structures can involve a nanometer-sized gap or a small metallic link bridging the two mesa features-thus Fano-type structures can be termed disconnected or connected.

**Fig. 4:**
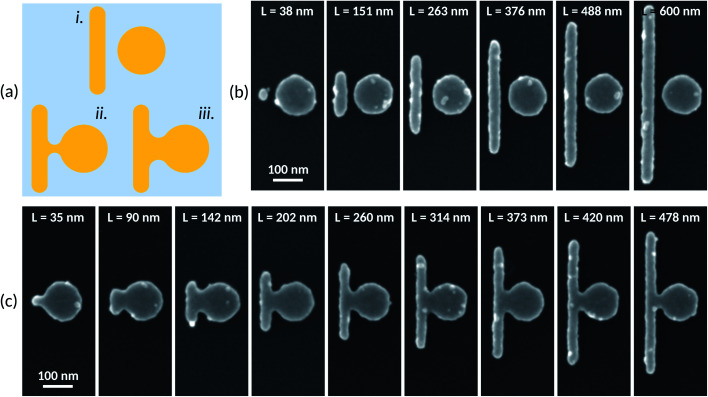
(a) Schematic representation of the electron beam lithographic patterning of the Fano-type structures. (i) Ideal patterning without overdosing or proximity effects; (ii) and (iii) show how the electron beam proximity effect can be harnessed to control the bridge in connected Fano-type structures whilst maintaining the shape and size of the individual disc and rod mesas. (b and c) Scanning electron microscopy images of the nanometer-sized, metallic Fano-type structures that we were able to create by harnessing the electron beam lithography proximity effect: (b) disconnected Fano-type nanostructures and (c) connected Fano-type structures.

The lateral size and gap width of the resulting metallic Fano-type structures are governed by the eBeam lithographic patterning—the evaporation process governs the thickness. Ideal patterning of such structures is shown schematically in Fig. 4a(i) (disconnected Fano-type structure). However, due to non-ideal dosing and proximity effects,^42^ such patterning of metallic mesas in close proximity is challenging to achieve in practice. In contrast, in order to gain control of the gap and bridge width of the Fano-type structures—whilst maintaining the shape and size of the disc and rod—here we have exploited the lithographic proximity effect^43–45^ to our advantage. Ultimately, the proximity effect (modified by dosing) between two patterns leads to a transition from a no bridge and a gap-like disconnected Fano-type structure somewhat like that shown in Fig. 4a(i) to connected structures shown in Fig. 4a(ii) and (iii).

The fabrication process of the nanostructures involves substrate cleaning, resist deposition by spin-coating, eBeam lithography, resist development, evaporation metallization, and lift-off. First, 170 μm thick glass substrates (Schott D 263®M Glass – Thorlabs) were cleaned using standard substrate cleaning methods (solvents, piranha solution)^46^ and dehydrated (180 °C for 10 minutes). Following this, a bilayer eBeam resist is deposited onto the glass wafer surface. The first resist layer is a 400 nm thick layer PMMA EL13% (MicroChem, USA) and is deposited onto the glass substrate by spin coating at 4500 rpm/1000 rpm s^−1^/15 s. A second resist layer (PMMA 950k diluted 5/3 Anisole, MicroChem, USA) is then deposited (4000 rpm/1000 rpm s^−1^/12 s) onto the first PMMA resist—this second resist layer will form the ‘overhang’ feature required for lift-off. The second resist layer has a thickness of 50 nm. Finally, a 5 nm-thick germanium layer is evaporated onto the resist surface to minimize charging problems during the eBeam writing. The bilayer resist-coated glass wafers are then patterned using eBeam in a commercial machine (Raith EBPG 500 Plus) at 100 kV, a dose current of 1000 pA, and a resolution of 1 nm. The Fano structural patterns were created with software (LayoutEditor)—the mask allowed the nanogap width (40–70 nm) and the nanorod length (38–624 nm) to be varied whilst the eBeam writing enabled the dose to be increased from 600 μC cm^−2^ to 50 μC cm^−2^ steps. After patterning, the Ge layer is removed in H_2_O_2_/H_2_O solution with a volume ratio of v/v = 1/1 for 60 s. The bilayer resist is then developed in a mixture of VLSI-grade methyl isobutyl ketone (MIBK) and isopropyl alcohol (IPA) with a volume ratio of v/v = 1/2 for 60 s to remove the exposed part of the resist and form the overhang and undercut features required for the lift-off of the metallization. The resist-patterned glass substrates are then deposited with gold (30 nm) is a commercial MEB 550S evaporation system (Plassys, France)—the evaporation was rotated but not tilted.^40,41^ Finally, the lift-off patterning of the metallization involves removing the bilayer resist using a commercial resist remover (Microchem, USA) at 70 °C for 2 h. Finally, the samples are rinsed in acetone and IPA and dried using dry nitrogen.

To observe the nanodisc/nanorod nanostructures using scanning electron microscopy (SEM), a thin germanium layer (3 nm) is deposited onto the substrate to ensure accurate imaging without charging effects – and subsequently removed as previously mentioned before the optical characterization. Fig. 4(b) and (c) show practical examples of the nanometer-sized, metallic Fano-type structures that we were able to create using our method of dose and dimension variation. Fig. 4(b) shows disconnected structures having a small gap. The gap width between the nanodiscs and the nanorods is evaluated to be 22.4 ± 4.8 nm. The disc diameter of the disconnected structures is 133 ± 1 nm. Fig. 4(c) shows connected Fano-type structures. The disc diameter of the connected structures is 134 ± 1.5 nm. As the nanorod length varies, the bridge neck width varies.

### Optical characterization

**Fig. 5:**
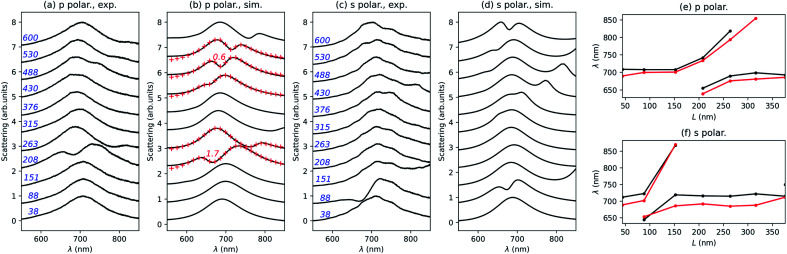
Comparison between the experimental, (a and c), and numerical, (b and d), scattering spectra of those disconnected structures in Fig. 4(b), for p, (a and b), and s, (c and d), polarizations. The nanorod length in nanometers is indicated in blue. Red crosses: fit with the coupled oscillator model, the value of *g*/*g*_c_ being given in red for *L* = 208 and 488 nm. Comparison between the experimental (black) and numerical (red) scattering maxima for the nanorod length up to 376 nm for p, (e), and s, (f), polarizations.

The optical scattering spectra have been measured using the setup presented by Eschimèse *et al.*,^32^ where the light beam is incident through a dark-field microscope objective under total internal reflection from the glass, and the scattered field is collected through the same microscope objective. Fig. 5 shows the comparison between experimental (a and c) and numerical (b and d) scattering spectra for p, (a and b), and s, (c and d), polarizations. The simulation has been done taking into account the experimental values of *L*, *R*, *w* and *g* of each nanostructure extracted from the SEM images.

A good agreement is found with some discrepancies attributed to the imperfections visible in the SEM images and inherent to the fabrication process. Consistently with the theoretical part, two anti-crossings are obtained for p polarization in the investigated parameter range of the nanorod length, close to *L* = 208 nm and *L* = 488 nm. In the experimental spectra, the second anti-crossing is less visible, which might again be related to the nanorod imperfections. The agreement between the experimental measurements and numerical simulations is clearly evidenced on the evolution of the wavelength associated with the scattering wavelengths for p (Fig. 5(e)) and s (Fig. 5(f)) polarizations. For p polarization, we have performed a fit of the numerical scattering spectra close to *L* = 208 and 488 nm. The obtained values for the normalized coupling coefficient *g*/*g*_c_ are respectively 1.7 and 0.6, consistent with a strong coupling between the ND bright mode and the NR second-order dark mode, and a weak coupling with the fourth-order dark mode. These results are as well in good agreement with the above numerical results.

**Fig. 6:**
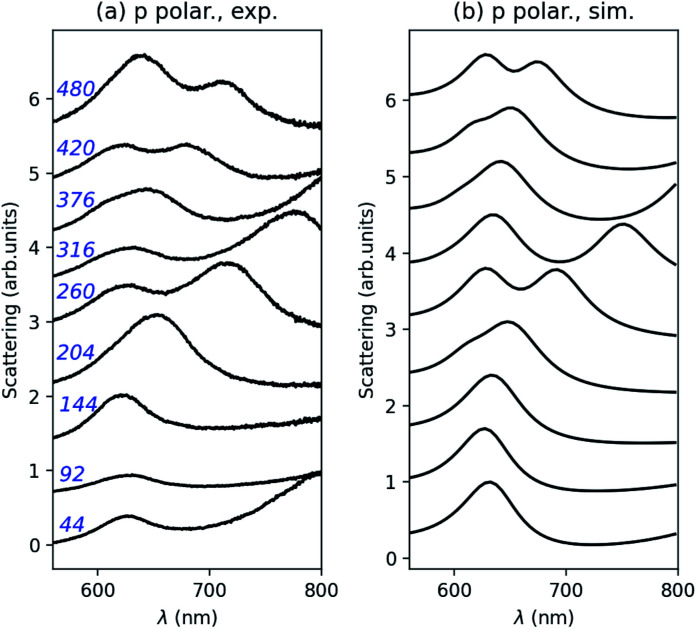
Comparison between the experimental, (a), and numerical, (b), scattering spectra of those connected structures in Fig. 4(c) for p polarization. The nanorod length in nanometers is indicated in blue.

Finally, experimental and numerical scattering spectra are compared for those connected structures in Fig. 6, in the case of p polarization. Again, the agreement between both is good despite the fabrication imperfection and variability of the width of the metal junction. According to numerical predictions, an almost *L* invariant peak is observed close to *λ* = 640 nm and two anti-crossings between the nanorod and nanodisk component modes occur close to *L* = 202 nm and *L* = 420 nm.

## Conclusion

We have explored numerically and experimentally the formation of hybridized modes between a bright mode supported by a gold nanodisk and either dark or bright modes of a nanorod, both elements being either separated by an air gap or connected by a metal junction. On the theoretical side, we have compared the simulated spectra and field distribution obtained under oblique-incidence plane wave illumination with quasi-normal mode computation using a home-made iterative method implemented in the open-source finite element method software FreeFem++. Varying the nanorod length, we evidenced in p polarization Fano profiles in the scattering spectra that dark modes associated with the nanorod have a resonance wavelength close to the nanodisk dipole mode, and strong coupling for a nanorod with a length close to 230 nm, provided that the gap is below 40 nm. Individual modes combine in hybridized bonding and anti-bonding modes which have been described in terms of field and surface charge distributions. These results are consistent with the set of parameters extracted from a fit of the extinction spectra with a coupled oscillator model, as for example the value estimated for the coupling parameter normalized to the losses reaches 1 for the same critical value of the gap. Finally, a metal connection between the nanorod and the nanodisk shows similar behavior to the disconnected system for s polarization but strongly modified modal properties for p polarization due to the redistribution of the surface charges close to the contact. The main consequence is that only anti-crossings are observed, at least up to 600 nm, when the resonance wavelength of the nanorod component approaches one of the disk components. Further investigations are required to elucidate the nature of the strongly interacting modes responsible for this behavior, for example by investigating the effect of a much longer metallic connection between the nanorod and the nanodisk. On the experimental side, we have successfully fabricated the investigated structures using top-down lithography methods. Proximity effects can lead to the formation of small metal junctions leading to the connected configuration, while careful compensation allows synthesised disconnected systems with a gap as low as 20 nm. We obtained a very good agreement between experimentally measured scattering spectra and numerical predictions. The methods and analysis presented in this work can be applied to a wide range of systems, for potential applications in light–matter interactions, biosensing or strain monitoring.^47,48^

## Conflicts of interest

There are no conflicts to declare.

## Supplementary Material

NA-004-D1NA00620G-s001

NA-004-D1NA00620G-s002

NA-004-D1NA00620G-s003

NA-004-D1NA00620G-s004
