# Supplementary material for: Strong and weak polarization-dependent interactions in connected and disconnected plasmonic nanostructures
Source: Nanoscale Adv. 2022 Jan 10;4(4):1173–81. doi: 10.1039/d1na00620g (PMC9417476; doi:10.1039/d1na00620g)
Supplement: NA-004-D1NA00620G-s004 [file NA-004-D1NA00620G-s004.pdf]

Electronic Supplementary Material (ESI) for Nanoscale Advances.
This journal is © The Royal Society of Chemistry 2022

Strong and weak polarization-dependent interactions in connected and disconnected plasmonic nanostructures Supplementary Information

Damien Eschimèse,[†,‡] François Vaurette,[†] Céline Ha,[†,¶] Steve Arscott,[†] Thierry Mélin,[§] and Gaëtan Lévêque[*,†]

†Univ. Lille, CNRS, Centrale Lille, Junia, Univ. Polytechnique Hauts-de-France, UMR 8520 - IEMN - Institut d'Electronique de Microélectronique et de Nanotechnologie, F-59000 Lille, France

‡Now at: Institut of Materials Research Engineering. 2 Fusionopolis Way, Singapore 138634

¶Now at: Univ. Grenoble Alpes, CEA, Leti, F-38000 Grenoble, France, France

§Univ. Lille, CNRS, Centrale Lille, Junia, Univ. Polytechnique Hauts-de-France, UMR 8520 - IEMN - Institut d'Electronique de Microélectronique et de Nasnotechnologie, F-59000 Lille, France

E-mail: gaetan.leveque@univ-lille.fr

1 Finite element simulations

Numerical simulations have been performed using two open-source softwares, namely FreeFem++ for the numerical resolution of Maxwell equations using FEM,[1–3] and Gmsh to create geometries and generate tetrahedral meshes.[4] For reasons of computer resources savings, symmetry (resp. anti-symmetry) conditions have been applied on the (Oxz) plane for p (resp. s) polarization. In order to prevent reflection of the scattered field on the boundaries of the simulation domain, an additional spherical PML (Perfectly Matched Layer) layer has been added around the simulation domain, delimited by the red dashed line. An example of geometry and mesh is shown on Fig. S1.

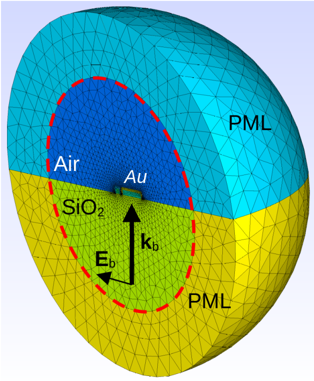

Figure S1: Geometry and mesh realized with Gmsh.

We have performed both plane-wave illumination and quasi-normal modes simulations. For that purpose, the dielectric constant for gold must be written under an analytical form in order to be able to extend it to complex frequencies. We have then fitted experimental data from Johnson and Christy[5] in the wavelength range between 500 nm and 900 nm with a two-oscillators Drude-Lorentz model, following :

$$\epsilon_D(k_0) = \epsilon_\infty \left(1 - \frac{\delta\epsilon(k_0)}{k_0^2}\right) \tag{1}$$

where $k_0 = \omega/c = 2\pi/\lambda$ is the wavenumber, and:

$$\delta\epsilon(k_0) = \frac{k_d^2/\epsilon_\infty}{1 + i\gamma_d/k_0} + \frac{s_l k_l^2}{1 + i\gamma_l/k_0 - k_l^2/k_0^2} \tag{2}$$

The obtained numerical values for the model parameters are listed in Table 1.

Table 1: Drude-Lorentz parameters, obtained by fitting Johnson and Christy data between 500 and 900 nm. Units are rad/nm, except for ϵ_∞.

ϵ_∞	$k_d(10^{-2})$	$\gamma_d(10^{-4})$	s_l	$k_l(10^{-2})$	$\gamma_l(10^{-3})$
5.115	4.415	2.817	0.3203	1.435	2.688

1.1 Planewave illumination

Simulations of the electromagnetic response of the system under monochromatic planewave illumination rely on the numerical resolution of the following equation :

$$\nabla \times \nabla \times \mathbf{E}_s - \epsilon_b(\mathbf{r}) k_0^2 \mathbf{E}_s = \left[\epsilon_D(k_0) - \epsilon_b(\mathbf{r})\right] k_0^2 \mathbf{E}_b \tag{3}$$

where the total electric field reads $\mathbf{E} = \mathbf{E}_b + \mathbf{E}_s$, with $\mathbf{E}_b$ the background field resulting from the reflection of the incident planewave on the glass interface (without the metal nanostructures), $\mathbf{E}_s$ the field scattered by the nanostructures, and $\epsilon_b(\mathbf{r})$ the position-dependent dielectric constant of the glass-air environment. This equation is then translated into its weak-form in order to be solvable with FEM. Details about the procedure to follow, including explanations about boundary conditions and finite-elements type can be found in reference.[6] Extinction, absorption and scattering cross-sections are respectively given by:

$$\begin{aligned}
C_{ext}(k_0) &= \frac{1}{n k_0 |\mathbf{E}_0|^2} \operatorname{Im} \int_V d\mathbf{r} \mathbf{P}(\mathbf{r}, k_0) . \mathbf{E}_b^*(\mathbf{r}, k_0), \\
C_{abs}(k_0) &= \frac{1}{n k_0 |\mathbf{E}_0|^2} \operatorname{Im} \int_V d\mathbf{r} \mathbf{P}(\mathbf{r}, k_0) . \mathbf{E}^*(\mathbf{r}, k_0), \\
C_{sca}(k_0) &= C_{ext}(k_0) - C_{abs}(k_0)
\end{aligned}$$

where Im stands for the imaginary part and the polarization vector is defined as:

$$\mathbf{P}(\mathbf{r}, k_0) = \left[\epsilon_D(k_0) - \epsilon_b(\mathbf{r})\right] \mathbf{E}(\mathbf{r}, k_0).$$

1.2 Quasi-normal modes

Quasi-normal modes are complex-frequency (or complex wavenumber) solutions of Eq. 3 without source term ($\mathbf{E}_b = \mathbf{0}$). This equation must then be rewritten in a shape allowing eigenvectors computation. Using 1, we obtain:

$$\frac{1}{\tilde{\epsilon}_\infty(\mathbf{r})} \nabla \times \nabla \times \mathbf{E}_s + \delta\tilde{\epsilon}(\mathbf{r}, \tilde{k}_0) \mathbf{E}_s = \tilde{k}_0^2 \mathbf{E}_s \tag{4}$$

where $\tilde{k}_0$ is the complex eigenvalue of the QNM, and:

$$\left.\begin{aligned} \tilde{\epsilon}_\infty(\mathbf{r}) &= \epsilon_\infty, \\ \delta\tilde{\epsilon}(\mathbf{r}, \tilde{k}_0) &= \delta\epsilon(\tilde{k}_0) \end{aligned}\right\}, \mathbf{r} \in V,$$

and:

$$\left.\begin{aligned} \tilde{\epsilon}_\infty(\mathbf{r}) &= \epsilon_b(\mathbf{r}) \\ \delta\tilde{\epsilon}(\mathbf{r}, \tilde{k}_0) &= 0 \end{aligned}\right\}, \mathbf{r} \notin V.$$

where V is the volume of the metal nanostructure. The real part of $\tilde{k}_0$ gives access to the resonance wavelength, $\lambda_0 = 2\pi / \operatorname{Re}(\tilde{k}_0)$, and the imaginary part to its width $\Delta\lambda_0 = \lambda_0 \operatorname{Im}(\tilde{k}_0) / \operatorname{Re}(\tilde{k}_0)$.

It is clear that any solution $\mathbf{E}_s$ of equation 4 for a particular $\tilde{k}_0$ is an eigenvector of the operator $1/\tilde{\epsilon}_\infty(\mathbf{r}) \nabla \times \nabla \times () + \delta\tilde{\epsilon}(\mathbf{r}, \tilde{k}_0)$ for the complex eigenvalue $\tilde{k}_0^2$. However, as this operator explicitly

and non-linearly depends on $\tilde{k}_0$, this equation is self-consistent and cannot be solved directly with the finite elements method. We choose instead to solve:

$$\frac{1}{\tilde{\epsilon}_\infty(\mathbf{r})}\nabla \times \nabla \times \mathbf{E}_s + \delta\tilde{\epsilon}(\mathbf{r}, k_e)\mathbf{E}_s = \tilde{k}_0^2\mathbf{E}_s \tag{5}$$

where k_e is a complex, close-enough estimate of $\tilde{k}_0$, which can be extracted for example from extinction spectra. Equation 5 is then a standard eigenvalue problem and can be solved with FreeFem++ using a generalized eigenvalue solver. The procedure to calculate the eigenmodes and eigenvalues is the following:

- compute extinction or absorption spectra under planewave illumination;
- extract estimates k_e of eigenvalues (real and imaginary parts) using wavelengths λ_e and widths $\Delta\lambda_e$ for maxima shown in absorption or extinction spectra: $k_e = 2\pi/\lambda_e(1+i\Delta\lambda_e/\lambda_e)$;
- solve Eq. 5 to obtain a new value of $\tilde{k}_0$;
- iterate k_e based on the obtained $\tilde{k}_0$ and repeat until convergence is reached.

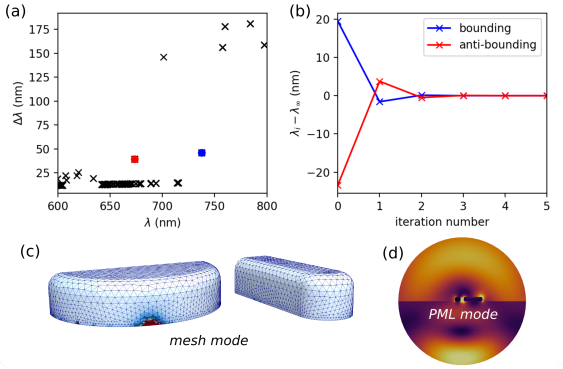

Figure S2: Numerical computation of plasmonic QNMs: (a) representation of the complex eigenvalues of equation 5 for a typical iteration; (b) convergency curves of the bonding and anti-bonding modes of the disconnected system for the standard geometrical parameters; (c) typical meshing (left) and PML (right) modes.

The main difficulty is to extract the physically meaningful $\tilde{k}_0$ among the very large number of eigenvalues of the operator projected on the basis of finite elements (see figure 2(a)), as most of the returned eigenvectors are modes either due to the meshing (figure S2(c)) or associated to the PMLs (figure S2(d)). Fortunately, efficient sorting can be performed on the imaginary part of $\tilde{k}_0$, which is generally comprised between low-losses mesh eigenmodes and large-losses PMLs eigenmodes. In practice, generally only two or three iterations are necessary to reach a converged eigenvalue with satisfactory precision, as shown on Fig. 2(b).

2 Uncoupled particles

The response of isolated ND and the NR is plotted on Fig. S3. The incidence plane contains the NR long axis. Both scattering and absorption spectra of the isolated gold nanodisk are dominated by a

broad resonance centered at 682 nm, and corresponds to the excitation of the in-plane dipolar mode, *(i)*. The width of that resonance, of about 100 nm, is related to the contribution of both internal dissipation and radiation losses. The NR is characterized by longitudinal bright and dark modes, with alternating positive and negative surface charges, bright modes being antisymmetric along the NR's long axis, while dark modes are symmetric. The 84-nm-long NR shows one resonance,

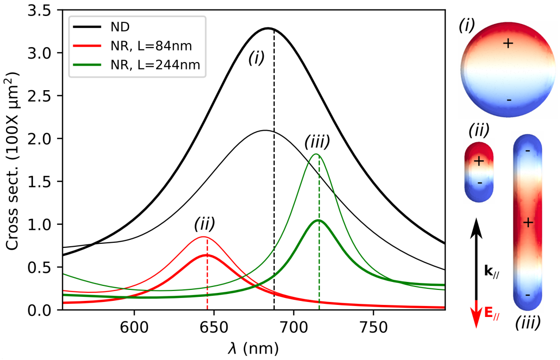

Figure S3: Scattering (thick lines) and absorption (thin lines) spectra of the ND and NRs with two different lengths L. The incidence plane contains the NR axis, and the polarization is p. The real part of the surface charges distribution is plotted for each associated QNM, whose wavelength is indicated by dashed lines.

(ii), close to 645 nm, corresponding to the lowest order bright mode, as evidenced by its surface charges distribution. The 244-nm-long rod shows a maximum close to 715 nm, and corresponds to the excitation of the lowest order dark mode, *(iii)*. Note that the NR's dark modes can only be excited because the illumination direction breaks the longitudinal symmetry of the nanoparticle. The maxima are close to the wavelengths obtained from QNM modes calculations, indicated by a vertical dashed line. The discrepancy is the largest for the ND dipole mode, with a value of about 6 nm, as QNM simulations leads to a dipolar mode wavelength of about 687.7 nm and a width $\Delta\lambda_0 = 114$ nm or $(\Delta\lambda_0)/\lambda_0^2 = 0.24\ \mu\text{m}^{-1}$, consistent with the spectra. For nanorods, the QNM wavelengths are 645.8 nm for the short NR ($\Delta\lambda_0 = 48$ nm or $(\Delta\lambda_0)/\lambda_0^2 = 0.12\ \mu\text{m}^{-1}$) and 716.0 nm for the long NR ($\Delta\lambda_0 = 37$ nm or $(\Delta\lambda_0)/\lambda_0^2 = 0.072\ \mu\text{m}^{-1}$). The widths are again consistent with the spectra, and we verify that it is larger for the bright mode than for the dark mode due to additional radiation losses.

3 Disconnected system

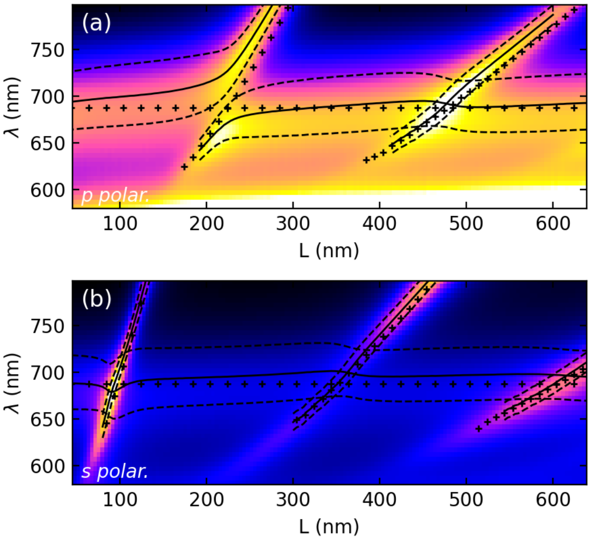

Figure S4: Numerical modeling of the disconnected system. (a): Absorption spectrum for p polarization, (b) for s polarization, compared with QNM wavelengths (solid lines) and widths (dashed lines).

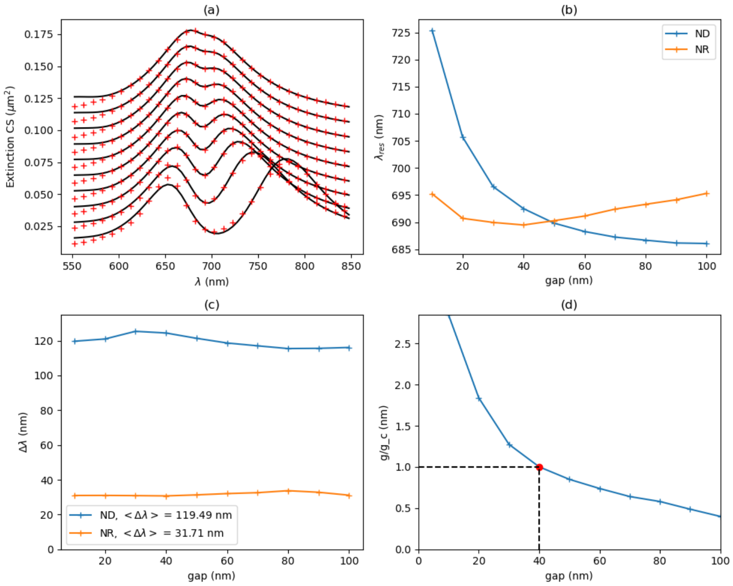

Figure S5: Comparison of the numerical scattering extinction spectra with the coupled oscillators model. (a) FEM simulations (solid black lines) and fit (red crosses), the gap being varied by step of 10 nm from bottom ($g = 10$ nm) to top ($g = 100$ nm). (b) Resonance wavelengths of the diabatic modes for ND (blue) and NR (orange). (c) Resonance widths of the diabatic modes for ND (blue) and NR (orange). (d) Evolution of the normalized coupling coefficient g/g_c with the gap ($g_c = \sqrt{\omega_0^2 - (\gamma_b - \gamma_b)^2/8}(\gamma_b - \gamma_b)/2$).

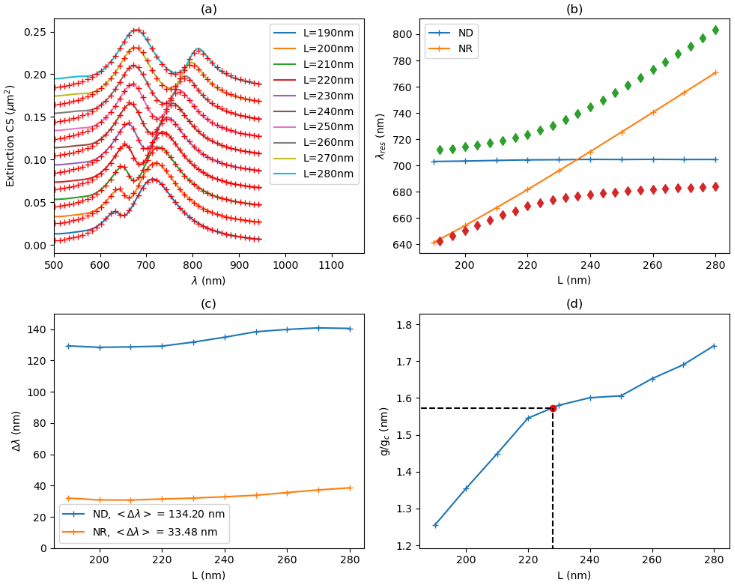

Figure S6: Comparison of the numerical scattering extinction spectra with the coupled oscillators model. (a) FEM simulations (solid lines) and fit (red crosses) for NR lengths close to $L = 228$ nm. (b) Resonance wavelengths of the diabatic modes for ND (blue) and NR (orange). (c) Resonance widths of the diabatic modes for ND (blue) and NR (orange). (d) Evolution of the normalized coupling coefficient g/g_c with the NR length.

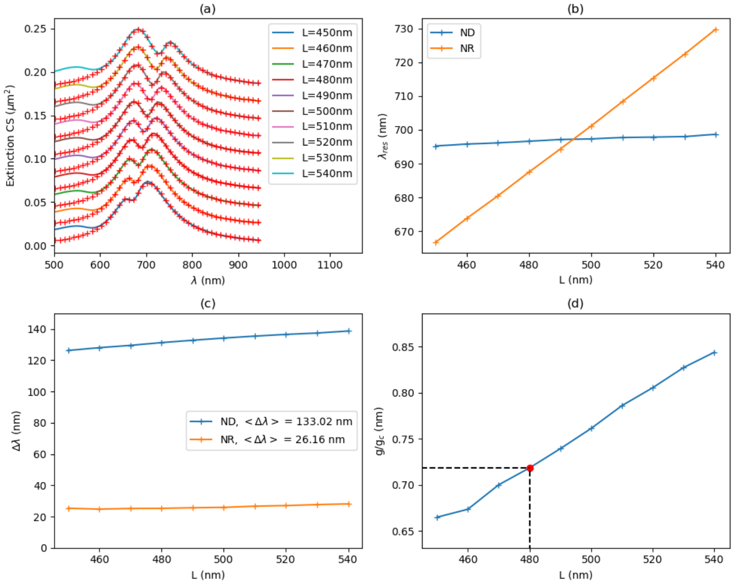

Figure S7: Comparison of the numerical scattering extinction spectra with the coupled oscillators model. (a) FEM simulations (solid lines) and fit (red crosses) for NR lengths close to $L = 480$ nm. (b) Resonance wavelengths of the diabatic modes for ND (blue) and NR (orange). (c) Resonance widths of the diabatic modes for ND (blue) and NR (orange). (d) Evolution of the normalized coupling coefficient g/g_c with the NR length.

4 Connected system

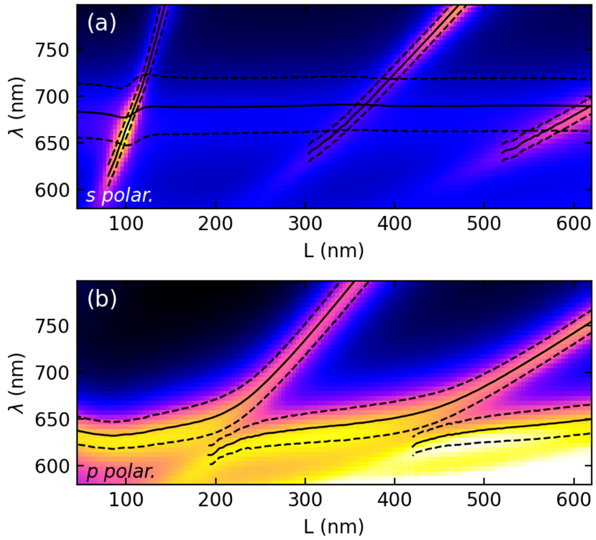

Figure S8: Numerical modeling of the connected system. (a): Absorption spectrum for s polarization, (b) for p polarization, compared with QNM wavelengths (solid lines) and widths (dashed lines).

References

(1) Hecht, F. New development in freefem++. *Journal of Numerical Mathematics* **2012**, *20*, 251–265.

(2) Mahi, N.; Lévêque, G.; Saison, O.; Marae-Djouda, J.; Caputo, R.; Gontier, A.; Maurer, T.; Adam, P.-M.; Bouhafs, B.; Akjouj, A. In Depth Investigation of Lattice Plasmon Modes in Substrate-Supported Gratings of Metal Monomers and Dimers. *The Journal of Physical Chemistry C* **2017**, *121*, 2388–2401.

(3) Zhang, S.; Bao, K.; Halas, N. J.; Xu, H.; Nordlander, P. Substrate-Induced Fano Resonances of a Plasmonic Nanocube: A Route to Increased-Sensitivity Localized Surface Plasmon Resonance Sensors Revealed. *Nano Letters* **2011**, *11*, 1657–1663.

(4) Geuzaine, C.; Remacle, J.-F. Gmsh: A 3-D finite element mesh generator with built-in pre- and post-processing facilities. *International Journal for Numerical Methods in Engineering* **2009**, *79*, 1309–1331.

(5) Johnson, P. B.; Christy, R. W. Optical Constants of the Noble Metals. *Physical Review B* **1972**, *6*, 4370–4379.

(6) Jin, J.-M. *Theory and computation of electromagnetic fields*; Wiley: Hoboken, N.J, 2010; OCLC: ocn548660179.
